# Four-Dimensional Dual-Energy Computed Tomography-Derived Parameters and Their Correlation with Thyroid Gland Functional Status

**DOI:** 10.3390/tomography11030022

**Published:** 2025-02-26

**Authors:** Max H. M. C. Scheepers, Zaid J. J. Al-Difaie, Nicole D. Bouvy, Bas Havekes, Alida A. Postma

**Affiliations:** 1 GROW Research Institute for Oncology and Reproduction, Maastricht University, 6229 ER Maastricht, The Netherlands; m.scheepers@maastrichtuniversity.nl (M.H.M.C.S.); z.al-difaie@maastrichtuniversity.nl (Z.J.J.A.-D.); n.bouvy@mumc.nl (N.D.B.); 2 Department of Surgery, Maastricht University Medical Center, 6229 HX Maastricht, The Netherlands; 3 Department of Internal Medicine, Division of Endocrinology and Metabolic Disease, Maastricht University Medical Center, 6229 HX Maastricht, The Netherlands; bas.havekes@mumc.nl; 4 NUTRIM School of Nutrition and Translational Research in Metabolism, Maastricht University, 6229 HX Maastricht, The Netherlands; 5 Department of Radiology and Nuclear Medicine, Mental Health and Neurosciences Research Institute (MHENS), Maastricht University Medical Center, P.O. Box 5800, 6202 AZ Maastricht, The Netherlands

**Keywords:** computed tomography (CT), four-dimensional CT (4D-CT), dual-energy CT (DECT), iodine concentration, thyroid function, head and neck imaging

## Abstract

Purpose: Dual-energy computed tomography (DECT) allows for the measurement of iodine concentration, a component for the synthesis of thyroid hormones. DECT can create virtual non-contrast (VNC) images, potentially reducing radiation exposure. This study explores the correlations between thyroid function and iodine concentration, as well as the relationship between thyroid densities in true non-contrast (TNC) and virtual non-contrast (VNC) images and thyroid function. Methods: The study involved 87 patients undergoing 4D-CT imaging with single and dual-energy scans for diagnosing primary hyperparathyroidism. Thyroid densities and iodine concentrations were measured across all scanning phases. These measurements were correlated with thyroid function, indicated by TSH and FT4 levels. Differences in thyroid density between post-contrast phases and TNC phases (ΔHU) were analyzed for correlations with thyroid function and iodine concentrations. Results: Positive correlations between iodine concentrations and TSH were found, with Spearman’s coefficients (R) of 0.414, 0.361, and 0.349 for non-contrast, arterial, and venous phases, respectively. Thyroid density on TNC showed significant positive correlations with TSH levels (R = 0.436), consistently across both single- (R = 0.435) and dual-energy (R = 0.422) scans. Thyroid densities on VNC images did not correlate with TSH or FT4. Differences in density between contrast and non-contrast scans (ΔHU) negatively correlated with TSH (*p* = 0.002). Conclusions: DECT-derived iodine concentrations and thyroid densities in non-contrast CT scans demonstrated positive correlations with thyroid function, in contrast to thyroid densities on VNC scans. This indicates that VNC images are unsuitable for this purpose. Correlations between ΔHU and TSH suggest a potential link between the thyroid’s structural properties to capture iodine and its hormonal function. This study underscores the potential value of (DE-) CT imaging for evaluating thyroid function as an additional benefit in head and neck scans.

## 1. Introduction

The current recommended method for the initial evaluation of thyroid function in patients involves the analysis of thyroid-stimulating hormone (TSH) levels in a blood sample [1,2]. TSH regulates multiple steps in the production of thyroid hormones. Thyroxine (T4) is the primary thyroid hormone present in the bloodstream. Assessments focused on free thyroxine (FT4) provide an accurate representation of thyroid gland performance, especially when conducted alongside TSH evaluations [1,3]. Iodine plays a vital role in the synthesis of thyroid hormones [4]. Consequently, the thyroid gland exhibits a relatively elevated iodine concentration, making it detectable on CT imaging due to iodine’s high electron density. This characteristic of the thyroid gland results in a notable CT attenuation value in non-contrast CT imaging studies [5].

Over the past decade, there has been a growing focus on the utilization of dual-energy computed tomography (DECT) for the diagnosis of head and neck conditions [6,7,8,9,10]. DECT involves the use of two distinct X-ray spectra, obtained from separate sources, a rapid kV switching system or a dual-layer spectral CT [11]. This method enables advanced tissue and material characterization, particularly for substances like iodine and water. One significant advantage of DECT scanning is its ability to generate virtual non-contrast (VNC) images from contrast-enhanced scans, potentially lowering radiation exposure by eliminating the need for a true non-contrast (TNC) phase [12]. Furthermore, DECT scanning may be beneficial in the assessment of thyroid function, given its capability for material decomposition [13].

At present, there is only limited data regarding the correlation between computed tomography (CT) density, as measured in Hounsfield units (HU), and the functional status of the thyroid gland, as assessed by TSH levels [14]. Previous research has established a link between the density of the thyroid gland observed in CT scans and the concentration of iodine within the gland [5,15]. It has been suggested that measuring the iodine concentration in the thyroid gland could be a useful method for evaluating the turnover of iodine, a key element in the metabolic pathway of thyroid hormones [16,17]. The application of dual-energy technology enables the measurement of iodine concentration within the thyroid gland via the material decomposition algorithm.

The primary aim of this study is to investigate the correlation between thyroid function and iodine concentration as measured with dual-energy computed tomography (DECT). Additionally, we aim to examine the relationship between thyroid densities from true non-contrast (TNC) and VNC images and thyroid function.

## 2. Methods

### 2.1. Patient Selection

This retrospective study included patients who underwent 4D-DECT imaging as part of the diagnostic process for primary hyperparathyroidism (PHPT) at Maastricht University Medical Center (MUMC+) in the Netherlands between 2017 and 2023. All patients were biochemically confirmed to have PHPT. Thyroid function was determined biochemically by measurements of thyroid stimulating hormone (TSH) and free thyroxine (FT4) concentrations. TSH and FT4 levels, measured in milli units per liter (mU/L) and obtained within one month of CT imaging, were retrieved from electronic patient records. Clinical and demographic data, as well as images, were retrieved from electronic patient records; the images were subsequently anonymized. The study protocol was approved by the Medical Ethical Commission (17-4-077.2—March 2017), and informed consent was waived.

### 2.2. Imaging Technique

4D-CT was performed on a third-generation dual-source CT scanner (Somatom Definition Force, Siemens, Erlangen, Germany). The imaging protocol consisted of: (1) a non-contrast scan, (2) a DECT post-contrast scan at 30 s (‘arterial’), and (3) a DECT 50 s post-contrast (‘venous’). Non-contrast imaging was performed using single energy CT acquisition (SECT) in 39 patients, and DECT acquisition in 48 patients. The post-contrast scans were conducted after administration of 70 mL Ultravist 300 (Bayer Schering) with a flow rate of 3 mL/s, followed by 30 mL saline flush at the same flow rate. Scan parameters were as follows: SECT scans were scanned in a care-kV energy mode. DECT at tube voltages 80/150 kVp and 35/23 Quality Reference mAs, CTDIVol 5.6 mGy, rotation time 500 ms, pitch 0.7, 1 mm slice thickness, FOV 270 mm, matrix, 512 × 512 and collimation 0.6. A soft tissue kernel was applied for all series. Mixed images were reconstructed, simulating a ‘120 kVp’ scan, with a weighted average of 0.5 between the two tube sources. DECT data were reconstructed with a quantification kernel (Q30). Iodine maps and VNC reconstructions were created using a three-material decomposition algorithm “Brain Hemorrhage” in a dedicated software package (Syngovia, version VB60, Siemens Healthcare) from non-contrast, arterial and venous phases. Iodine concentrations were measured in millimoles per liter (mmol/L).

### 2.3. Image Analysis

Thyroid densities, measured in Hounsfield units (HU), were recorded within a circular region of interest (ROI) with a maximum diameter of 20 mm^2^ during all scanning phases (0, 30, and 50 s) on a PACS viewing station (Sectra, Linkoping, Sweden). In our study, the ROI was carefully positioned to ensure it encompassed homogeneous thyroid parenchyma while avoiding visible confounders such as thyroid nodules (e.g., cystic or solid lesions), blood vessels, and imaging artifacts (e.g., streaks or beam hardening). A standardized, fixed-size circular ROI was used (maximum diameter of 20 mm^2^), and placement was guided by visual inspection to ensure it encompassed only homogeneous thyroid parenchyma. All ROI placements were reviewed and confirmed by a board-certified neuroradiologist to ensure accuracy and consistency. The iodine concentration in thyroid tissue was calculated using a three-material decomposition algorithm (“Brain Hemorrhage”) on a dedicated workstation (Syngovia VB60, Siemens, Erlangen, Germany) for 0, 30, and 50 s phase images. The three-material decomposition algorithm calculates the iodine concentration by decomposing the attenuation values into three base materials: iodine, blood (hemorrhage), and soft tissue. By leveraging the different attenuation coefficients of these materials at low and high kiloelectron volt (keV) energy spectra, the algorithm can accurately quantify iodine concentration within a region of interest, even in the presence of complex surrounding structures. To evaluate thyroid density differences, we calculated the changes between post-contrast phases—arterial (ΔHUa) and venous (ΔHUv)—and non-contrast (TNC) densities. Differences were determined by subtracting the TNC value from the arterial phase value (ΔHUa—TNC) and the venous phase value (ΔHUv—TNC), respectively. These calculations provide measures of arterial enhancement at 30 s (ΔHUa) and venous enhancement at 50 s (ΔHUv).

### 2.4. Statistical Analysis

Patient characteristics were summarized using mean and standard deviation for continuous variables, and count and percentage for categorical variables. The Spearman rank correlation coefficient was employed to assess correlations between thyroid function and thyroid densities (HU) across all scanning phases. Additionally, this coefficient was also used to evaluate the relationship between thyroid function and iodine concentration in DECT measurements. Statistical tests were performed separately for different scanning protocols, which included SECT non-contrast scans and DECT non-contrast scans. Statistical analyses utilized SPSS software (version 27; IBM-SPSS, Inc., Chicago, IL, USA), with two-tailed tests and significance set at *p* ≤ 0.05.

## 3. Results

A total of 87 patients, comprising 16 males and 71 females, were included with a mean age of 63.7 ± 10.9 years. Among the patients, 86.2% (*n* = 75) were euthyroid, with one patient (1.1%) diagnosed with hyperthyroidism and 11 patients (12.6%) diagnosed with hypothyroidism. The mean TSH level was 2.1 ± 1.2 mU/L, and FT4 levels, measured in 35 patients, averaged 15.7 ± 3.0 mU/L. The mean thyroid tissue densities in non-contrast, arterial, and venous CT images were 103.5 ± 25.1 HU, 185.7 ± 39.1 HU, and 149.9 ± 25.9 HU, respectively. The mean thyroid densities for VNC at 30 s and VNC at 50 s were 48.4 ± 15.4 HU and 50.1 ± 15.2 HU, respectively. The mean iodine concentration with DECT was 1.6 ± 1.1 mmol/L in non-contrast scans, 4.7 ± 1.7 mmol/L in arterial scans, and 3.5 ± 1.4 mmol/L in venous scans. The patient characteristics are detailed in Table 1.

### 3.1. Correlation Between Thyroid Density and Thyroid Function: Thyroid Density on Non-Contrast CT Scans (SECT + DECT (Mixed)) and TSH/FT4

**Figure 1:**
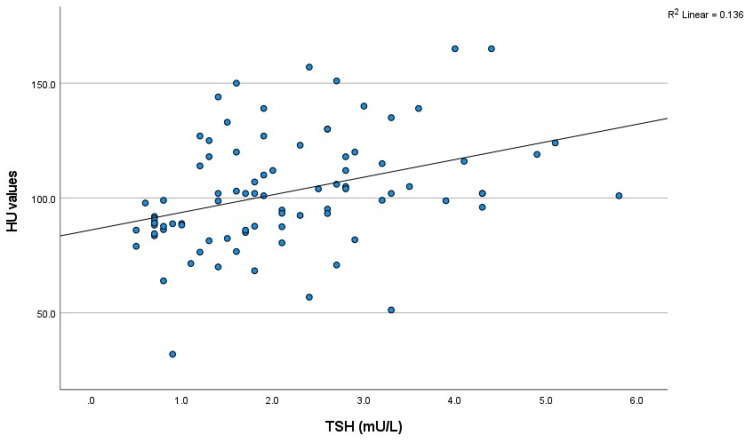
The scatter plot of the relationship between non-contrast HU value and thyroid function. (TSH) (*n* = 87). HU: Hounsfield unit; TSH: thyroid stimulating hormone.

A positive correlation was observed between thyroid density in non-contrast CT scans and TSH levels (*p* < 0.001, R = 0.436) (Figure 1 and Table 2). A non-significant negative correlation was found between thyroid density in non-contrast CT scans and FT4 levels (*p* = 0.078, R = −0.302).

***i. SECT: Thyroid Density and TSH/FT4***

**Figure 2:**
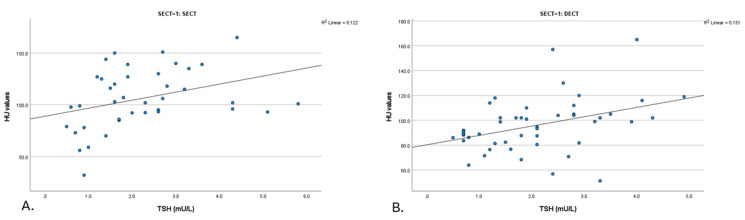
Scatter plot of the relationship between HU value and thyroid function (TSH) for SECT and DECT images. (**A**). TSH (SECT) (*n* = 39) (**B**). TSH (DECT) (*n* = 48). DECT: dual-energy computed tomography; HU: Hounsfield unit; SECT: single-energy computed tomography; TNC: true non-contrast; TSH: thyroid stimulating hormone; VNC: virtual non-contrast.

A significant positive correlation was identified between thyroid density and TSH levels in a subgroup of 39 patients with SECT non-contrast scans (*p* = 0.006, R = 0.435) (Figure 2). In this group, a negative correlation was observed between the thyroid density values from these scans and FT4 levels, although it was not statistically significant (*p* = 0.169, R = −0.389) (Table 3).

***ii. DECT (mixed): Thyroid Density and TSH/FT4***

On DECT non-contrast scans (mixed) (*n* = 48), significant positive correlations were observed between thyroid density and TSH levels (*p* = 0.003, R = 0.422) (Figure 2). No significant correlation was detected between the thyroid density values from these scans and FT4 levels (*p* = 0.406, R = −0.191) (Table 3).

***iii. VNC Reconstructions: Thyroid Density and TSH/FT4***

The thyroid densities on VNC at 30 and 50 s demonstrated no significant correlation with either TSH or FT4 levels (Table 3).

### 3.2. Thyroid Density Changes (ΔHU): Correlations with TSH

**Figure 3:**
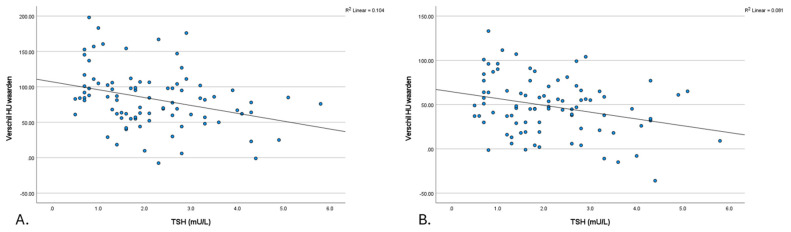
The scatter plot of the relationship between the ΔHU-TNC value and thyroid function (TSH). (**A**). Δ (HU **arterial**—TNC) value and thyroid function (TSH) (**B**). Δ (HU **venous**—TNC) value and thyroid function (TSH). HU: Hounsfield unit; TNC: true non-contrast; TSH: thyroid stimulating hormone.

A negative correlation was observed between differences in thyroid density between post-contrast (e.g., arterial and venous) and non-contrast (TNC) densities and TSH levels. Figure 3 presents a scatter plot that illustrates the correlation between the changes in Hounsfield units (ΔHU, calculated as post-contrast minus TNC) in thyroid density and TSH levels. This is shown separately for arterial enhancement at 30 s (a) ΔHUa (R = −0.321, *p* = 0.002) and enhancement in the venous phase at 50 s (b) ΔHUv (R = −0.222, *p* = 0.039).

### 3.3. Iodine Concentration in Thyroid: Correlation with TSH

**Figure 4:**
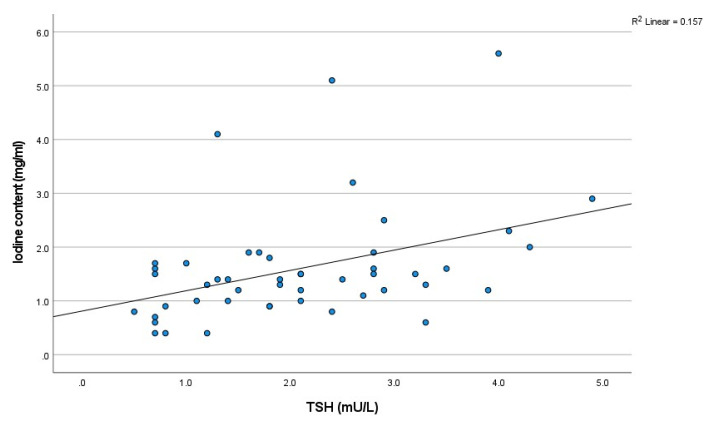
The scatter plot of the relationship between iodine concentration on TNC and thyroid function (TSH) (*n* = 48). TSH: thyroid stimulating hormone.

The iodine concentrations observed during all scanning phases (non-contrast, arterial, and venous) demonstrated a positive correlation with TSH levels. Spearman’s rank correlation coefficients (R) were 0.414 for the non-contrast phase, 0.361 for the arterial phase, and 0.349 for the venous phase. The correlations were all statistically significant, with *p*-values of 0.003, <0.001, and <0.001, respectively (Figure 4).

## 4. Discussion

This study identified a significant positive correlation between thyroid density on non-contrast CT scans and TSH levels. However, no significant correlations were observed between thyroid density and FT4 levels. Additionally, differences in thyroid density between post-contrast and TNC images were negatively correlated with TSH. Positive correlations were also observed between thyroid iodine concentration and TSH levels in all contrast phases. In contrast, VNC reconstructions showed no significant correlations with either TSH or FT4 levels.

Prior studies have investigated the correlations between the thyroid density on CT and thyroid function [5,14,18,19]. The results of our study demonstrated a significant positive correlation between thyroid densities on non-contrast CT scans and TSH levels. These findings are consistent with previous research, underscoring the potential of CT imaging in evaluating thyroid function [8,18,20,21]. Conversely, we observed a negative correlation between thyroid density and FT4 levels; however, this finding did not reach statistical significance. In a comparable cohort to our study, predominantly characterized by normal TSH levels, Pandey et al. identified a similar significant positive correlation between thyroid density and TSH values (R = 0.400, *p* < 0.001) [14]. In contrast, Li et al. found no significant correlation between thyroid density and TSH in a relatively small cohort of 43 patients. The relatively small sample size in the latter study likely contributed to considerable variability in the analysis. Additionally, Li et al. examined the correlations between FT4 and thyroid density but did not identify any significant correlations, which is consistent with the findings of this study. This discrepancy in correlations between thyroid density and TSH versus FT4 may be attributed to their distinct physiological roles. Previous literature has suggested that TSH may directly affect thyroid structure and activity, with potential correlations detectable on CT scans [22]. In contrast, FT4 levels, influenced by factors like protein binding and hormone conversion [23], do not necessarily correlate with structural changes visible on CT, making this correlation less evident.

A negative correlation was observed between differences in thyroid density in post-contrast images (e.g., arterial and venous) and TSH levels. The difference in Hounsfield units (HU) between post-contrast and TNC images represents a valuable metric for assessing the thyroid gland’s ability to capture the iodinated contrast material. Essentially, a greater uptake of contrast by the thyroid gland may be associated with lower TSH levels, indicating a potential relationship between the gland’s structural characteristics and its hormonal function.

One of the advantages of DECT is its capability to generate virtual non-contrast images, which have the potential to remove the TNC phase. This feature potentially eliminates the need for the TNC phase, thereby reducing the radiation exposure to patients. To our knowledge, this study is the first to explore the correlation between thyroid density on VNC images and thyroid function. Our results showed that VNC images did not exhibit significant correlations with either TSH or FT4 levels. This finding underscores the challenges of using VNC images, as the material decomposition algorithm removes both intrinsic and contrast-derived iodine, potentially reducing sensitivity to structural or functional thyroid variations. As a result, TNC images, which retain intrinsic iodine, may be more appropriate for assessing thyroid function in clinical settings. The thyroid’s naturally higher iodine concentration causes it to appear denser on TNC images than surrounding tissues and pathologies such as parathyroid adenomas [24]. Both the injected as well as the intrinsic iodine of the thyroid are removed by the material decomposition algorithm, making the thyroid appear less dense on VNC images. This potentially reduces the effectiveness of VNC imaging for detailed analysis of (para) thyroid pathologies [25], stressing the need for TNC images.

Another key advantage of DECT scanning is its ability to perform material characterization and calculation of concentration [26,27]. In our study, we measured iodine concentration within the thyroid gland. A positive correlation was found between the iodine concentration and TSH levels. Our study also noted a non-significant negative correlation between the iodine concentration in non-contrast CT scans and FT4 levels. In comparison, a previous study by Li et al. did not observe significant correlations between iodine concentration and TSH and/or FT4 levels [21]. However, despite the smaller sample size in their study, Li et al. did find a significant positive correlation between iodine concentration and FT3. Based on this finding, they proposed that measuring thyroid iodine concentration with DECT could be useful for assessing human iodine nutritional status and evaluating thyroid function. This could potentially improve the diagnosis, detect non-symptomatic patients and impact the management of thyroid diseases.

In accordance with the aforementioned findings, another study employed cervical spine CT scans, which were initially scanned for different purposes, in order to assess thyroid function. The authors of this study propose that monitoring incidental low thyroid densities and conducting follow-up functional tests may be beneficial, as it avoids the additional costs and radiation exposure typically associated with such procedures [14]. Additionally, DECT is emphasized as an effective method for assessing thyroid iodine levels, aiding in the diagnosis and treatment of iodine-related conditions and monitoring hyperthyroidism treatment outcomes, highlighting its importance in thyroid health [21].

The current study underscores the potential of CT imaging as an additional method for thyroid function assessment in head and neck scans. The use of both SECT and DECT scans demonstrated significant correlations between thyroid density and TSH levels in non-contrast scans, highlighting the consistency and reliability of single- and dual-energy CT imaging. Additionally, incorporating these imaging-based assessments into routine head and neck scans could provide a non-invasive, incidental opportunity to screen for thyroid dysfunction in at-risk populations. By identifying patients with abnormal ΔHU values or iodine uptake patterns early, clinicians can implement timely interventions, potentially improving outcomes and reducing the burden of thyroid-related complications.

However, the limitations of this study, such as its retrospective design and small sample size, warrant caution in generalizing the results. Furthermore, the study did not account for the potential dependency of VNC image accuracy on variations in iodine background concentrations. Future research should investigate how differing iodine concentrations, particularly in high-uptake tissues like the thyroid gland, may impact the reliability of VNC imaging for assessing thyroid structure and function. Expanding the scope to include thyroid antibody measurements, such as thyroid peroxidase antibodies (TPOAb) and thyroglobulin antibodies (TgAb), to better understand the interplay between iodine concentration, thyroid autoimmunity, and thyroid function could also provide deeper insights into the relationship between iodine concentration and thyroid function, further enhancing the clinical utility of CT imaging in thyroid health assessment.

In conclusion, the potential link between the thyroid’s structural properties for iodine uptake and its hormonal function was reflected by the positive correlation of (DE-) CT-derived iodine concentrations, thyroid densities in non-contrast CT scans and ΔHU with thyroid function. This suggests that (DE-) CT imaging may be employed to evaluate thyroid function as an additional benefit in head and neck scans. However, further research is needed to fully understand the clinical significance of the correlations between thyroid densities, iodine concentration, and thyroid function.

## Figures and Tables

**Table 1:** Patient demographics of study cohort.

	All Patients (*n* = 87)	TNC—SECT (*n* = 39)	TNC—DECT (*n* = 48)
Age years (SD)	63.7 (10.9)	62.0 (11.9)	65.1 (9.9)
Female % (N)	81.6 (71)	84.6 (33)	79.2 (38)
TSH mU/L (SD)	2.1 (1.2)	2.2 (1.3)	2.1 (1.1)
FT4 mU/L (SD)	15.7 (3.0)	15.6 (3.5)	15.7 (2.7)
Euthyroid % (N)	86.2 (75)	76.9 (30)	93.8 (45)
Hyperthyroidism % (N)	1.1 (1)	0 (0)	2.1 (1)
Hypothyroidism % (N)	12.6 (11)	23.1 (9)	4.2 (2)

**Table 2:** Correlation between thyroid function and HU values on SECT and DECT TNC images and DECT-derived iodine concentration. DECT: dual-energy computed tomography; FT4: thyroxine; HU: Hounsfield unit; SECT: single-energy computed tomography; TNC: true non-contrast; TSH: thyroid stimulating hormone; VNC: virtual non-contrast. *: Indicates statistical significance (*p* < 0.05).

	TNC (*n* = 87)	TNC—SECT (*n* = 39)	TNC—DECT (*n* = 48)
TSH mU/L	2.1 (1.2)	2.2 (1.3)	2.1 (1.1)
Spearman’s Rho	0.436	0.435	0.422
*p*-value	<0.001 *	0.006 *	0.003 *
**FT4** mU/L	15.7 (3.0)	15.6 (3.5)	15.7 (2.7)
Spearman’s Rho	−0.302	−0.389	−0.191
*p*-value	0.078	0.169	0.406
**Iodine concentration** mmol/L			1.6 ± 1.1
Spearman’s Rho	-	-	0.745
*p*-value	-	-	<0.001 *

*: Indicates statistical significance (*p* < 0.05).

**Table 3:** Correlation between thyroid density (HU) on VNC images (derived from 30 and 50 s post-contrast phases) and thyroid function (TSH/FT4). FT4: thyroxine; HU: Hounsfield unit; TSH: thyroid stimulating hormone; VNC: virtual non-contrast.

	VNC 30	VNC 50
Thyroid density HU (SD)	48.4 (15.4)	50.1 (15.2)
**TSH** mU/L	2.1 (1.2)	2.1 (1.2)
Spearman’s Rho	−0.034	0.015
*p*-value	0.755	0.894
**FT4** mU/L	15.7 (3.0)	15.7 (3.0)
Spearman’s Rho	0.288	0.138
*p*-value	0.093	0.429

## Data Availability

The datasets produced or examined in the course of this study have been incorporated into the published article.
